# Effect of a herbal preparation including nanoencapsulated thymol, carvacrol and cinnamaldehyde in feed on production parameters in the farrow-to-wean sector

**DOI:** 10.2478/jvetres-2025-0058

**Published:** 2025-10-21

**Authors:** Jarosław Wojciechowski, Dominika Siuda, Małgorzata Juszkiewicz, Ruud Schrijver, Javier Banuls Soto, René Bonekamp, Chandra Pareek, Zygmunt Pejsak, Grzegorz Woźniakowski

**Affiliations:** Lecznica dla Zwierząt (Private veterinary practice), 86-300 Grudziądz, Poland; Department of Diagnostics and Clinical Sciences, 87-100 Toruń, Poland; Interdisciplinary Doctoral School Academia Copernicana, 87-100 Toruń, Poland; Department of Virology and Animal Viral Diseases, National Veterinary Research Institute, 24-100 Puławy, Poland; Animal Health Concepts - AHC, 8141 Heino, the Netherlands; Department of Infectious, Invasive Diseases and Veterinary Administration, Institute of Veterinary Medicine, Faculty of Biological and Veterinary Sciences, Nicolaus Copernicus University in Toruń, 87-100 Toruń, Poland; University Veterinary Medicine Centre, University of Agriculture in Krakow, 31-120 Kraków, Poland

**Keywords:** biocide, growth performance, nutritional intervention, phytogenic compound, weaning piglets

## Abstract

**Introduction:**

Plant extracts have been noted to be effective against multiple bacterial and viral pathogens. The aim of the study was to determine the effect of a preparation based on nanoencapsulated thymol, carvacrol and cinnamaldehyde administered in the feed to farrowing sows on piglet production parameters.

**Material and Methods:**

The farrowing group unit had a stocking density of 1,100 pens, divided into groups of 25 stalls. The experiment was carried out in a group of 168 sows. The animals were selected and placed in the farrowing house in a randomised procedure. The animals were divided into two equal groups (84 animals in each), a control group and an experimental group. Appropriate biosecurity rules were applied and adhered to for each stage of production.

**Results:**

The results showed positive effects of the supplement in feed in terms of average birth weight of litters and of piglets and number of piglet days fed by the sow. The experimental group of farrowing sows showed more effective feed consumption, better condition of the sows during weaning of the piglets, more even litters in terms of health and a significantly lower number of piglet deaths.

**Conclusion:**

The use of the preparation positively influenced the number of piglets sold, with 88.5% reaching the market in the experimental group compared to 82.6% in the control group. In addition, fewer abortions and less lameness were observed in the experimental group than in the control group. The piglets born of control group sows performed worse economically than piglets born of experimental group sows, requiring feeding or being sold underweight.

## Introduction

Long-standing studies of natural plant extracts extracted from fruits, herbs, algae and lichens have demonstrated their potential inhibitory effects on the *in vitro* and *in vivo* replication of bacteria, viruses and fungi pathogenic to humans and animals (2, 8, 9, 11, 12, 16, 23, 24, 26–31). In view of the trends to reduce the use of antibiotics because of rising bacterial resistance and to address human and animal health as indivisible or One Health, natural plant extracts have also found use as an alternative to antimicrobials. Extract use can reduce the proliferation of pathogenic bacterial microflora including *Yersinia* spp., *Streptococcus* spp., *Salmonella* spp. and *Candida* spp. (14, 15, 20, 26, 34). Because One Health prioritises the reduction of synthetic antimicrobial use, opportunities for drug substitution with phytogenic compounds are valuable. Scientific reports have indicated that plant extracts are effective in inhibiting the proliferation of herpes simplex, hepatitis A, severe acute respiratory syndrome, infectious bronchitis, avian influenza, Newcastle disease and porcine epidemic diarrhoea viruses and feline coronavirus (2, 3, 9, 16, 25, 27). In addition, it has been shown that natural compounds produced by many plant species can replace synthetic antiviral analogues, including acyclovir, ribavirin and remdesivir (4, 13). These compounds may mainly stop viral proliferation by blocking specific protein receptors on susceptible cells to prevent virus attachment (33, 34). Other mechanisms of action of natural compounds with biocidal activity are blocking the expression of proteins and production of enzymes crucial during integration of the viral envelope and inhibiting the production of viral proteins responsible for transcription, translation and further processing of viral RNA, DNA or proteins (9, 18, 21).

A very common cause of diarrhoea in newborn piglets and piglets at weaning is infection with pathogenic intestinal flora, *Salmonella* spp. among them, from the contaminated environment or from farrowing sows (1, 7, 17, 24). Many of the microflora pathogenic to piglets are resistant to antibiotics or resistant to physicochemical agents. The use of nanoencapsulated essential oil components such as thymol, carvacrol and cinnamaldehyde has shown promising results in animal production. These phytochemicals possess well-documented antimicrobial, antioxidant and anti-inflammatory properties, which contribute to improved gut health, immune modulation and pathogen control. Nanoencapsulation enhances their physicochemical stability and protects the active compounds from degradation in the gastrointestinal tract, enabling a controlled release and increasing their bioavailability. As a result, these compounds can more effectively exert their biological effects, which improve growth, reduce mortality and support better overall health in livestock. Phytochemical achievement of growth optimisation aligns with current trends toward sustainable and antibiotic-free animal production. The aim of this feed-supplementation study was to determine the effect on reproduction parameters of preparation containing nanoencapsulated thymol, carvacrol and cinnamaldehyde, when given to farrowing sows. In addition, the effects of the product on the incidence of lameness and abortion in sows and the number of piglets and sows sold were evaluated. The product was tested under field conditions. The observed parameters suggest that the product may be a valuable tool for lowering production costs by improving reproductive performance and animal health.

## Material and Methods

### Experimental design: animals, diets and sampling

The experiment was carried out in a group of 168 sows. The animals were selected and placed in the farrowing house in a randomised way. The study was carried out in one farrowing house, which allowed the same environmental conditions to be maintained for the entire experimental group. All animals were subjected to examination concurrently to eliminate temporal variability and were provided with the same access to water and feed. The maximum housing stocking density was 25 pigs, and sows were kept in identical single pens. The animals were divided into equal control (C) and experimental (Ex) groups. Gilts accounted for 12% of group C and for 8% of group Ex. From the day of farrowing to weaning, sows from both groups received the same feed twice a day at fixed times. Sows in group C received feed without additives, while in group with supplementation was added to the standard feed ration. The sows in the experimental group had the supplementation of tested preparation in their feed ration increased weekly according to the following scheme: in the first week the daily supplement was 12 g (6 g per feeding), in the second week it was 20 g (10 g per feeding) and in the third and fourth weeks 26 g was given daily (13 g per feeding).

### Measurement of production parameters

The following production and health parameters were recorded: total number of piglets born, average total litter and piglet weight at birth, duration of lactation (days), number of piglets weaned per sow, average total litter and piglet weight at weaning and sow body condition at entry into the farrowing unit. Litter health statistics noted were the number of piglets treated for clinical conditions and the number of piglets removed from the herd at sale for health or welfare reasons. The rate of oestrus onset and pregnancy rate were determined as reproductive parameters. Standard biosecurity measures were implemented at all stages of production, including adherence to an all-in, all-out management system. When clinically indicated, antibiotic therapy was administered individually *via* intramuscular injection, with booster doses applied as necessary.

## Results

The results obtained with preparation show its positive effect for use as a supplement in feed when evaluating production parameters such as average litter birth weight, average piglet birth weight and number of piglet days fed by the sow. The number of piglets born, the number of piglets weaned, the weaning weight of the litter and the average weaning weight of piglets, and the number of piglets treated including those with diarrhoea were not seen to be under any positive effect of the preparation (Table 1). However, subjective evaluation indicated better feed consumption, better condition of the sows during weaning of the piglets and more even litters in terms of health in the experimental group of farrowing sows, where a significantly lower number of piglet deaths occurred. This was confirmed by the data presented below: in group C the number of live-born piglets was 1,163, of which 56 were not sold because they were removed from the herd at sale because of poor health or welfare. The final sold number of piglets of sows in this group was 961 (82.6% of live births). Piglet mortality in the control group reached 17.4%. In comparison, in group Ex the number of live-born piglets was 1,094 and 7 piglets were not sold. The final sold number of piglets of experimental sows was 969. Piglet mortality in the experimental group was 11.4% (Table 2).

**Table 1.** Production parameters of piglets from the born of control (C) and experimental (Ex) group sows fed with given plain feed or feed supplemented with preparation based on nanoencapsulated thymol, carvacrol and cinnamaldehyde

Parameter	Group C	Group Ex
Number of piglets born	1,290	1,190
Number of live piglets born	1,163	1,094
Average litter birth weight (kg)	22.23	20.28
Average piglet birth weight (kg)	1.26	1.31
Number of feeding days	26	25
Number of weaned piglets	1017	976
Average litter weaning weigh (kg)	77.45	74.14
Average piglet weaning weight (kg)	5.88	5.96
Number of pigs treated	28	30
Number of litters treated for diarrhoea	20	20

**Table 2.** Piglet production parameters born of control (C) and experimental (Ex) group sows given plain feed or feed supplemented with nanoencapsulated thymol, carvacrol and cinnamaldehyde

Parameter	Group C	Group Ex
Number of live piglets born	1,163	1,094
Number of piglets euthanised	56	7
Number of piglets sold	961	969
Mortality (%)	17.4	11.4

One sow died in group C, and three were euthanised because of lameness. Sale was necessary of 13 of the 84 sows in the group, 5 sows had “empty” oestrus and 4 of them had abortions. In the experimental group, there were no losses among those 84 sows, 2 of them were euthanised because of lameness and 10 of them were sold. Two sows had “empty” oestrus. Additionally, 1 sow did not farrow, probably because of an insemination error. Mean parameter values were determined for group C and group Ex. The results are shown in Table 3.

**Table 3.** Production parameters of sows from the control (C) and experimental (Ex) groups given plain feed or feed supplemented with nanoencapsulated thymol, carvacrol and cinnamaldehyde

Parameter	Group C	Group Ex
Sow deaths	1	0
Sows euthanised because of lameness	3	1
Number of sows sold	13	10
Sows with “empty” oestrus	5	2
Abortions	4	0
Older sows/sows of high parity	3	6
Low body condition score/lameness	1	2

## Discussion

Natural plant extracts and pro-and prebiotics (10, 24) represent an alternative to the application of antibiotic treatment or application of ZnO in cases of diarrhoea in piglets (5–7). Several effective biocidal substances are currently being investigated which may replace antibiotics and contribute to better production rates in the piglet rearing (1, 17, 31, 32). Many of the new biocidal substances can be encapsulated so that they can be administered in drinking water or with feed, allowing them to be transported to the intestines with maximal bioavailability and reducing diarrhoea in piglets during the first days of life and after weaning (20). The most common causes of neonatal porcine diarrhoea are infections with enterotoxigenic strains of *E. coli*, porcine reproductive and respiratory syndrome virus, transmissible gastroenteritis virus, porcine epidemic diarrhoea virus, rotaviruses, anaerobes such as *Clostridium perfringens* types A and C, *Enterococcus* spp. and coccidia (6, 27). The presented results of the application of a nanoencapsulated preparation with thymol, carvacrol and cinnamaldehyde delivered in feed showed a positive impact in terms of more profitable production parameters in piglets. A previously conducted study by Ahmed *et al*. (1) administering pure citric acid found no significant improvement in production parameters in piglets, because the growth parameters and intestinal microbial environment were not significantly affected. However, some immunity parameters were positively changed. On the other hand, acidification of feed for weaning piglets with pure organic acids and their mixtures would be expected to improve growth performance (1, 14, 32). Positive effects of the application of probiotics were observed in reproductive performance–related parameters in sows, including an increase in the number of piglets, higher piglet growth rates and greater body weights (11, 17, 19, 24, 31). Similarly to these findings, our experiment showed better feed consumption in farrowing sows, better body condition during weaning of the piglets, more even litters and a significantly lower number of piglet deaths. The positive effect of nanoencapsulated natural compounds including mixtures of thymol, carvacrol and cinnamaldehyde may be explained by the antioxidant activity of these preparations, which confirms numerous previous investigations (19, 22, 23, 27, 33). The positive effect of the tested product, the applied additive, was evident when analysing the numbers of live-born piglets, the final number of piglets sold and piglet mortality. In group Ex, the number of live-born piglets was 1,094, 7 piglets were not sold and 969 were sold after piglet mortality at 11.4%. These parameters indicated a considerably more profitable herd than the control sows. Application of the natural extracts contained within this preparation also showed positive effects on the production parameters of the sows in the zero mortality in the experimental group animals and the improvement of other production parameters.

## Conclusion

High piglet diversity makes it difficult to form equal groups of weaned piglets, and, consequently, to sell a batch of animals for slaughter at the same time. The application of the tested preparation raised the proportion of piglets sold (88.5%) over that of the control group (82.6%), and a lower mortality rate was observed in piglets born to its recipients (11.4%) than in those with unsupplemented dams (17.4%). In addition, fewer abortions and less lameness were observed in the experimental group than in the control group sows. Group C sows’ piglets performed worse as profit generators (*e.g*. in requiring feeding or in selling underweight) than group Ex sows’ offspring. In addition, lower piglet mortality and a higher percentage of all piglets born being fit for sale were observed. In the future, further studies are planned on a larger number of pigs in other production groups including fattening pigs and boars.
